# Aflatoxin Regulations and Global Pistachio Trade: Insights from Social Network Analysis

**DOI:** 10.1371/journal.pone.0092149

**Published:** 2014-03-26

**Authors:** Travis R. Bui-Klimke, Hasan Guclu, Thomas W. Kensler, Jian-Min Yuan, Felicia Wu

**Affiliations:** 1 Department of Environmental and Occupational Health, University of Pittsburgh, Pittsburgh, Pennsylvania, United States of America; 2 Department of Biostatistics and Public Health Dynamics Laboratory, University of Pittsburgh, Pittsburgh, Pennsylvania, United States of America; 3 Department of Pharmacology, University of Pittsburgh, Pittsburgh, Pennsylvania, United States of America; 4 Department of Environmental Health Sciences, Johns Hopkins Bloomberg School of Public Health, Baltimore, Maryland, United States of America; 5 Department of Epidemiology, University of Pittsburgh, Pittsburgh, Pennsylvania, United States of America; 6 Department of Food Science and Human Nutrition and Department of Agricultural, Food and Resource Economics, Michigan State University, East Lansing, Michigan, United States of America; Umeå University, Sweden

## Abstract

Aflatoxins, carcinogenic toxins produced by *Aspergillus* fungi, contaminate maize, peanuts, and tree nuts in many regions of the world. Pistachios are the main source of human dietary aflatoxins from tree nuts worldwide. Over 120 countries have regulations for maximum allowable aflatoxin levels in food commodities. We developed social network models to analyze the association between nations’ aflatoxin regulations and global trade patterns of pistachios from 1996–2010. The main pistachio producing countries are Iran and the United States (US), which together contribute to nearly 75% of the total global pistachio market. Over this time period, during which many nations developed or changed their aflatoxin regulations in pistachios, global pistachio trade patterns changed; with the US increasingly exporting to countries with stricter aflatoxin standards. The US pistachio crop has had consistently lower levels of aflatoxin than the Iranian crop over this same time period. As similar trading patterns have also been documented in maize, public health may be affected if countries without aflatoxin regulations, or with more relaxed regulations, continually import crops with higher aflatoxin contamination. Unlike the previous studies on maize, this analysis includes a dynamic element, examining how trade patterns change over time with introduction or adjustment of aflatoxin regulations.

## Introduction

Aflatoxins, produced by the foodborne fungi *Aspergillus flavus* and *A. parasiticus,* primarily contaminate food crops such as maize, peanuts, and tree nuts in tropical and subtropical regions of the world [1]. These crops are often subject to poor storage conditions, which favor aflatoxin accumulation [2], [3]. It has been estimated that over 5 billion people worldwide are exposed to dietary aflatoxins [2].

Among the most potent naturally occurring liver carcinogens known, “naturally occurring mixes of aflatoxins” (e.g., aflatoxins B1, B2, G1, G2) have been classified by the International Agency for Research on Cancer as a Group 1 human carcinogen [4]. The risk of developing liver cancer in individuals exposed to chronic hepatitis B virus (HBV) infection and aflatoxin are up to 30 times greater than the risk in individuals exposed to either risk factor alone [5], and there appears to be a multiplicative relationship between aflatoxin and HBV in inducing liver cancer [6].

Worldwide, over 120 countries have regulations for aflatoxins in food as of 2003, the last year in which the Food and Agriculture Organization (FAO) of the United Nations compiled aflatoxin regulatory standards [7]. This is an increase of 30% compared to 1995 [8] in terms of number of countries with aflatoxin maximum levels (MLs). These regulations are meant to protect human health by decreasing dietary exposures to aflatoxin [7], [9]. Several of these regulations are summarized in **Table 1**. In addition to the increase in the total number of countries regulating mycotoxins, the number of commodities/products that are being regulated on aflatoxin contamination levels within each country has also increased from 1995 to 2003.

**Table 1:** Summary of aflatoxin regulations (total aflatoxins) for pistachios in selected countries.

Country	Standard for total allowable aflatoxins in 1995 [8] (ng/g)	Standard for total allowable aflatoxins in 2003 [7] (ng/g)
USA	15	15
Iran	No Regulation	15
European Union (EU)	No Regulation	4 (Changed to 10 in 2009)
Belgium	No Regulation	4 (Changed to 10 in 2009)
Canada	15	15
Germany	4	4 (Changed to 10 in 2009)
Hong Kong	15	15
Japan	20*	20*
Saudi Arabia	No Regulations	No regulations
China	No Regulations	No Regulations
Egypt	No Regulations	No Regulations
The Netherlands	10*	4 (Changed to 10 in 2009)
Russia	5	5

*Japan and The Netherlands have (had) a standard for AFB_1_ only. AFB_1_ represents about half of the sum of total aflatoxins (AFB_1_+ AFB_2_+ AFG_1_+ AFG_2_); thus, the maximum allowable level of AFB_1_ was doubled.

While many regulations on maximum allowable aflatoxin levels are put in place to protect human and animal health, they may also have substantial impacts on food trade activities around the world [10]. Because of the large number of countries that have regulations on allowable mycotoxin levels in imported foodstuffs, there has been recent interest in whether associations exist between regulations and trade. Wu and Guclu [9], [11] recently examined aflatoxin regulations in a network of global maize trade and found that nations tend to trade maize with other nations that have identical or very similar aflatoxin standards, even defying geographical distances to engage in such trade.

The goal of this paper is to examine the impact of aflatoxin regulations on trade patterns for pistachios worldwide. Pistachios are the main contributor to dietary aflatoxin exposure from tree nuts, accounting for 7–45% of humans’ total aflatoxin exposure from all sources [12]. Aflatoxin contamination events in pistachios have commonly disrupted trade in the last two decades. In 1997, the European Union (EU) banned all pistachio imports from Iran due to aflatoxin levels ranging from 11–400 ng/g in pistachio consignments intended for European import. In 2002, the United Kingdom called for a reinstatement of the 1997 ban on Iran pistachios due to aflatoxins contaminating over 10% of sampled consignments. Most recently, in 2010, the US instituted a ban on all Iran pistachios.

The global pistachio market is dominated by Iran and the United States; over 70% of the world’s pistachio exports come from Iran (47%) and the US (25%) [13]. However, there appears to be a difference in the crop quality between countries with Iran pistachios containing an average of 54 ng/g aflatoxin, and the majority of US pistachios containing average levels below the EU standard of 10 ng/g [12].

The history of pistachio contamination with aflatoxin combined with the market domination by Iran and the US make it feasible for trade patterns to be analyzed over time to determine if associations exist between pistachio crop quality, exports and global trade. Using social network modeling tools [14], we tracked global trade patterns from the US and Iran each year between the years of 1996 and 2010, inclusive, to determine if aflatoxin regulations in the pistachio-importing nations worldwide appear to play a role in whether nations import primarily from the US or Iran, independent of other political factors. Each model contains information about the volume of trade of pistachios as well as aflatoxin regulation data for each country. Network modeling has provided a useful tool for other public health applications, including prediction models for disease transmission and control [15], [16], [17], prediction of obesity and smoking in social groups [18], [19], and modeling global maize trade [9], [11].

We hypothesized, based upon an earlier study examining the impact of EU aflatoxin regulations on US pistachio and almond trade [9], [20], that the nations with the strictest standards would import from countries with the highest quality crop in order to reduce economic losses. If this hypothesis holds true, public health is likely being negatively affected in many ways. As shown in Wu & Guclu [9], countries with similar regulations trade more maize with each other than countries with dissimilar regulations. If this pattern also exists for pistachios on a global scale, it may also exist for a wide range of commodities. Therefore, countries without regulations may be importing more contaminated commodities from other countries with lenient or no regulations, predisposing individuals in those nations to higher risk of adverse effects associated with food contaminant exposures.

## Methods

### Social Network Modeling

**Figure 1:**
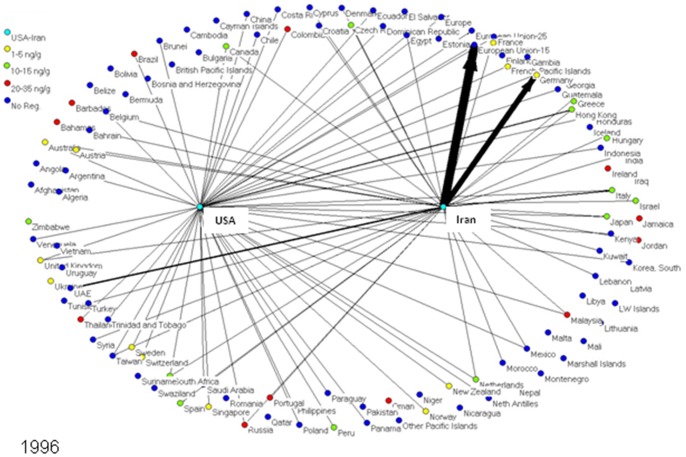
Social network models of pistachio trade are shown for selected years. 1996 serves as an initial time point for the global pistachio trade dataset.

To determine market trends in pistachio trade, a social network model was created for every year from 1996 to 2010, inclusive. Each model depicts the amount of pistachios exported from the US and Iran to each importing nation worldwide. Each nation is represented as an individual node or “actor” in the network models, connected to other nations by lines (edges) if these two nations traded pistachios (one nation exporting to the other). In these specialized directed social network models - one for each year from 1996–2010 - the US and Iran are the two central nodes exporting pistachio to other countries, and the amounts of pistachio exports are represented by the thickness of the line in the network representations. The nodes on the boundary of the graphical network representations signify the countries, which are importing pistachios from either the US or Iran or both **(** **Figures 1** **–** **5** **)**.

**Figure 2:**
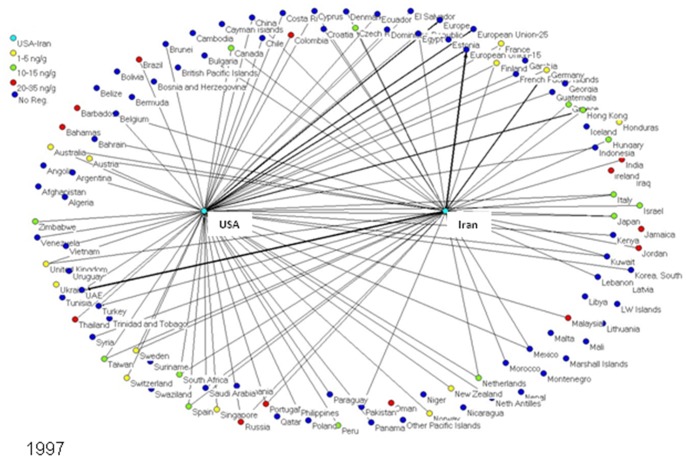
In 1997, Iranian pistachios were contaminated with extremely high levels of aflatoxin that led to the EU’s banning of pistachio imports from Iran for that year.

**Figure 3:**
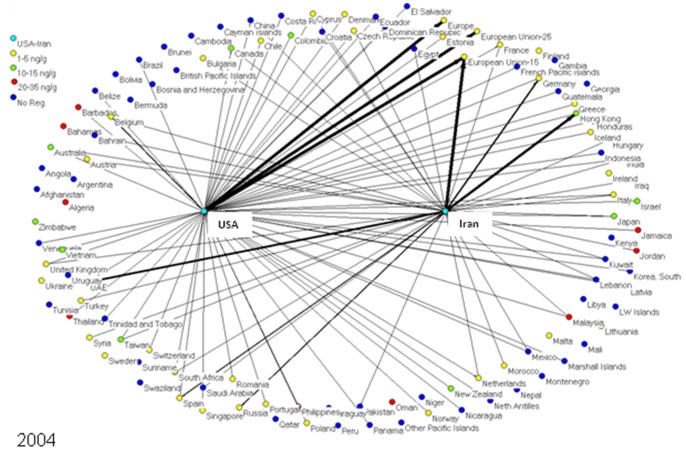
In 2004, global pistachio trade patterns shifted the year after the EU implemented a harmonized 4/g aflatoxin standard in tree nuts.

**Figure 4:**
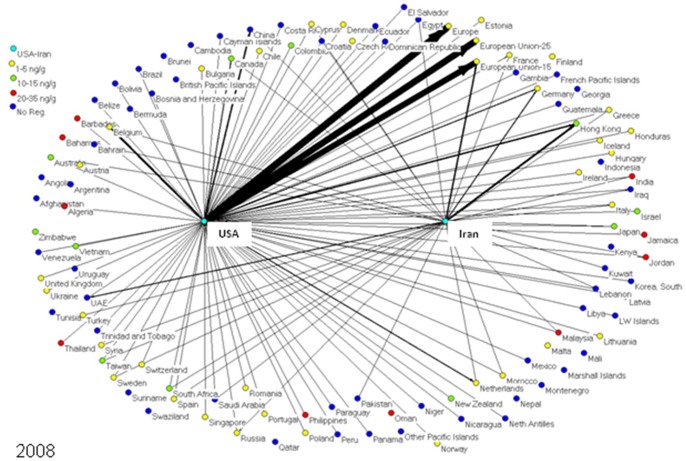
2008 is the first year the US exported more pistachios than Iran, and the final year before the EU relaxed the aflatoxin regulation for tree nuts to 10/g.

**Figure 5:**
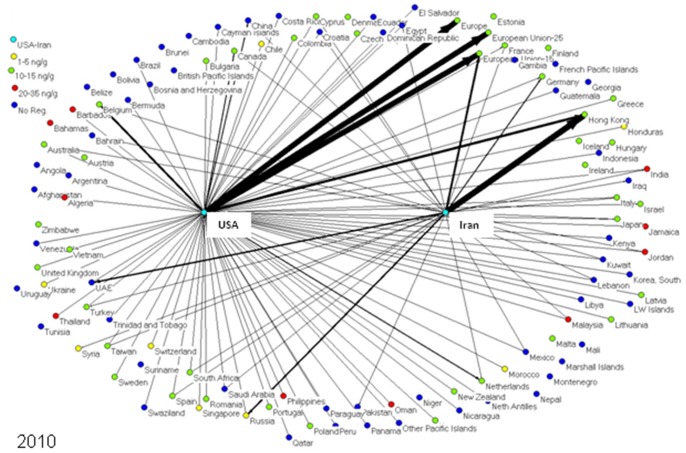
2010 shows the most recent available trends in pistachio trade. In 2009, the EU relaxed its tree nut aflatoxin standard from 4/g to10 ng/g.

The nodes representing the pistachio-importing nations are color-coded according to the strictness of their aflatoxin regulations; i.e., maximum tolerable level of total aflatoxins (aflatoxin B1+B2+G1+G2) in pistachios. As aflatoxin regulations and the amount of exports changed over the years, the colors of nodes and line thickness also changed.

The network modeling software Pajek [21] was used to create the 15 yearly network models. Pistachio export data was compiled using the USDA Foreign Agricultural Service Global Agricultural Trade System (FAS GATS - http://www.fas.usda.gov/gats/default.aspx) and the Iran Pistachio Association (IPA - http://www.iranpistachio.org/). Data on the number of pistachio consignment rejections in the EU was compiled using the EUROPA - Rapid Alert System for Food and Feed (RASFF) - (http://ec.europa.eu/food/food/rapidalert/index_en.htm).

The models were analyzed longitudinally to determine the main importers from the US and Iran; as well as to determine the association, if any, between aflatoxin standards and pistachio trade patterns over the years. The total amount of pistachios exported from the US and Iran was compared year by year to determine the major pistachio exporter in each model. Additionally, the amount of pistachios and aflatoxin regulatory level was compared between years to determine trends in exports. The amounts of pistachio exports from the US and Iran to countries which had changes in aflatoxin tree nut regulations were analyzed to determine if countries with strict standards imported from Iran or the US, or if there were no differences. Likewise, this analysis was done for pistachio-importing nations with relaxed or no aflatoxin standards on pistachios.

### Crop Quality Assessment

The relative levels of aflatoxin contamination in the US and Iranian pistachio crops were compared in two ways. First, governmental reports, peer-reviewed publications, and online agricultural databases were searched for information regarding aflatoxin levels in US and Iranian pistachios. Next, the RASFF database was used to determine the number of rejected consignments being exported from the US and Iran to the EU. The number of rejected consignments from each country was graphed along with the amount of pistachios imported from the US and Iran. As the RASFF database also reports contamination levels measured in rejected food lots, it was possible to calculate average aflatoxin levels in rejected pistachio consignments entering the EU from both the US and Iran.

### Market Segregation Analysis

We analyzed market segregation due to competition between USA and Iran in two ways. First, the United States’ export share over that of Iran was calculated for the top ten pistachio importers worldwide. The proportion of the US exports was graphed for each of the top ten countries for each year, using the equation:
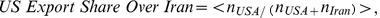
where *n*_USA_ represents the amount of pistachios exported from the US to a particular country, *n*_Iran_ represents the amount of pistachios exported from Iran to the same country, and <…> represents an average over the top ten importers. To calculate the US’s export share over that of Iran, the amount of pistachios exported from the US to each of the top ten countries was divided by the total amount exported to each country from the US and Iran. A proportion of 1 signifies that 100% of pistachios imported to a particular country came from the US, whereas a proportion closer to 0 signifies that the majority of pistachios were imported from Iran.

Second, in order to take into account each importing country’s aflatoxin standard, the weighted average for each country’s imports was assessed longitudinally. The inverse of each country’s standard (a measure of strictness of the aflatoxin standard) was multiplied by the amount of pistachios imported to obtain a strictness-weighted amount. The inverse of each standard was used so that a stricter standard was associated with a higher score, while nations with no aflatoxin standards for pistachios were given a strictness score of zero. This analysis was used to determine if the US or Iran traded with more countries with stricter aflatoxin standards. Because the US dataset contained more countries than Iran, the datasets were matched, each containing 41 countries with pistachio export data. The following equation was used:
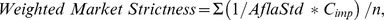
where AflaStd represents the aflatoxin standard of the importing country, *C*_imp_ represents the amount of pistachios imported from the US or Iran, and *n* represents the total number of importing countries.

Finally, to serve as a control in order to determine if segregation was a result of aflatoxin regulations or of political factors unrelated to aflatoxin, we conducted the same analyses using grape exports. Grapes, which are not commonly contaminated by aflatoxin, are not subject to aflatoxin regulations. It was assumed that any sanctions placed on Iran would be followed by all UN nations. Greece is a member of the UN. Therefore, Iran’s grape exports were compared to Greece’s grape exports (a country with similar amounts of exports) to a variety of countries. Any difference in grape trading patterns between Greece and Iran with other countries could infer the impact of sanctions – or any type of non-aflatoxin-related barriers – on Iran’s exporting business activities.

## Results

**Figures 1** **–** **5** show five of the fifteen network models created of pistachio exports from the US and Iran to various nations worldwide. In 1996 (**Figure 1**), Iran was the major exporter of pistachios to all countries, with over 120,000 tons of pistachios exported; compared to only 22,000 tons for the US. The EU-15 was the major importer of pistachios in 1996 with 89,000 tons imported; 96% of which came from Iran. The US’ largest exports went to Hong Kong, the EU and Canada; the three countries made up 65% of the United States’ exports. As for Iran, 68% of pistachio exports went to the EU while 13% went to the United Arab Emirates (UAE).

In 1996, 71 out of 113 pistachio-importing countries did not have aflatoxin regulations for pistachios. Three of the top 5 importers from Iran did not have aflatoxin regulations; this included the EU, which did not have a blanket regulation for all member countries. Hong Kong and Canada, the US’ major pistachio importers, each regulated aflatoxin at 15 ng/g [8]. Thirteen countries had strict regulations ranging from 1–5 ng/g, fourteen had moderate regulations of 10–15 ng/g, and thirteen had the least strict regulations at 20–34 ng/g [8]. Most importantly, no aflatoxin regulation existed for Iran in 1995, while the US regulated total aflatoxins at 15 ng/g [8]. This did not change until 2003 when Iran began to regulate total aflatoxins in pistachios at 15 ng/g [8].

Due to high levels of aflatoxin contamination in Iranian pistachios in 1997, Iran’s pistachio exports in that year (**Figure 2**) decreased by 46%, whereas the US increased total pistachio exports by 17%. Globally, Iran remained the major exporter of pistachios over the US; however, the EU imported only 23% of its crop from Iran in 1997, compared with 96% the year before. Yet Iran’s major importer in 1997 remained the EU (30% of total Iranian exports), while the UAE imported 28% of Iran’s total crop. The US continued to export the majority of its pistachios to Hong Kong (34%), the EU (33%) and Canada (7%). No major changes in aflatoxin regulations occurred between 1996 and 1997.

Between the years 1998 and 2003, Iran remained the top exporter of pistachios globally and to the EU. In the year following the 1997 aflatoxin outbreak, the EU nearly quadrupled its pistachio imports from Iran while also increasing imports from the US. Over the next four years, Iran’s major importers remained the EU, UAE, and Hong Kong. For the US, Japan, Hong Kong, and the EU were the major importers with Mexico importing increasing amounts of pistachios starting in 2000.

Over this time, regulations also began to change. By 2003, the EU had imposed a harmonized aflatoxin standard in tree nuts, including pistachios, of 4 ng/g. This regulation went into place for all EU member states, as well as the candidate member states in 2003. Regardless of the aflatoxin regulation in the EU, Iran has remained the top producer and exporter of pistachios globally for most of the years since 1996. In 2003, the total Iranian export amount topped 160,000 tons with over 70% exported to the EU (21%), UAE (36%), and Hong Kong (13%). Just over 35,000 tons of pistachios were exported from the US in 2003, the second highest total to date. The EU imported 65% of the US’ pistachio crop, while Hong Kong only imported 2%, choosing Iran as their main supplier. Japan and Canada remained main importers of US crops and China increased its imports from 29 tons in 1996 to over 3100 tons in 2003.

**Figure 3** shows 2004 exports from Iran and the US a year after the significant changes were made for aflatoxin standards in the EU. From 2003 to 2004, Iran total exports dropped 30%, while the US increased exports about 36%. Shortly after the EU aflatoxin regulations came into place, there was a significant decrease in Iranian pistachio exports to the EU and UAE; however, there were substantial increases in Iranian exports to Russia, Iraq and Hong Kong. Canada and Japan remained major importers of US pistachios in 2004, but were joined by the UK and UAE.

From 2004 to 2008 (**Figure 4**), major changes occurred in pistachio trade with little or no changes occurring in pistachio regulations. For the first time, the US was the major exporter of pistachios with exports reaching over 120,000 tons, almost 40,000 more than Iran. The EU imported nearly 60,000 tons of pistachios from the US, compared to only 13,500 from Iran. The top 5 importers of US pistachios in 2008 were the EU, China, Hong Kong, Mexico and Canada. China and Mexico have no aflatoxin regulations; however, Hong Kong and Canada regulate aflatoxin at 15 ng/g and the EU at 4 ng/g.

In 2009, the EU revised the aflatoxin standard in tree nuts to a more relaxed standard of 10 ng/g. Results of this change are shown in **Figure 5**. As of 2010, Iran has regained the lead in global pistachio exports over the US, with over 160,000 tons exported. Their major importers were Hong Kong (38%), the EU (11%), and the UAE (10%). While the EU was a top importer of Iranian pistachios, the amount of pistachios imported from the US was nearly triple this amount. Major importers of US crops, other than the EU (43%), included Mexico (3%), Japan (3%), China (6%) and Canada (7%).

Overall, these network models and the associated analyses show that the US and Iran have exported to different markets over the past 15 years. Notable changes in trade occurred after the EU instituted stricter aflatoxin standards. The US is trading more with countries with stricter standards; however, Iran has kept total exports up by exporting to countries with less strict regulations.

**Figure 6:**
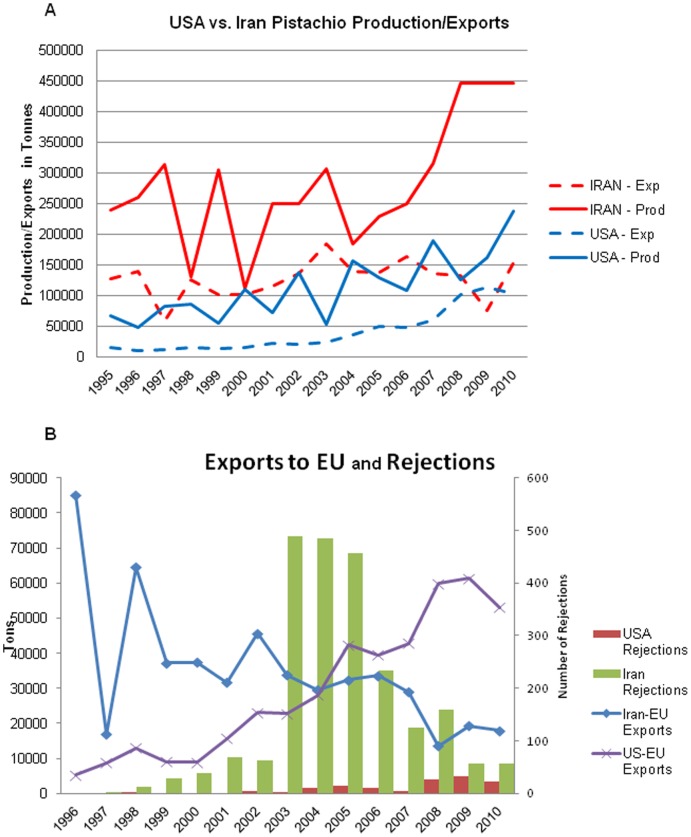
Pistachio production and crop quality comparisons between the US and Iran. **A)** Iranian and US pistachio production and exports between 1995 and 2010. Both the US and Iran have high and low production years over the 15 year period. Both countries are showing increased production over the past 10 years. Iran once dominated exports; however, the US has closed the gap and exported a larger quantity of pistachios than Iran in 2009 [23]. **B)** EU-specific exports from the US and Iran and number of pistachio consignment rejections. US to EU exports have trended upwards since 1997, whereas Iran to EU exports have decreased. The number of rejections of Iranian pistachios has remained higher than of US pistachios since the induction of the RASFF system in 1997. Iran pistachio rejections peaked in the mid-2000s, but are decreasing as the total export amount to the EU is decreasing.

**Figure 6A** summarizes the amount of pistachios produced and exported by the US and Iran over the past 15 years. Over this time, Iran has remained the top producer and exporter of pistachios; however, US pistachio production and exports are slowly trending upwards. Iran had noticeable drops in both production and exports in the years 1997–1998 and 2000, largely due to excessively high aflatoxin levels. Over this same time period, the US has continued to increase pistachio exports such that it now roughly matches Iranian pistachio exports, although overall, US production is still much lower.

To determine the relative quality of Iranian and US pistachios in terms of aflatoxin levels, the number of RASFF rejections for aflatoxins exceeding the EU regulation was calculated between 1997 and 2010. No data prior to 1997 were available. The number of RASFF pistachio consignment rejections in the EU were graphed alongside the amount of pistachios imported from the US and Iran in **Figure 6B**. The amount of pistachios exported from the US to the EU is slowly on the rise, whereas EU imports from Iran have been decreasing. The number of rejections of Iranian pistachios peaked in 2003 with 489 and was followed closely in 2004 with 485. US rejections peaked at 32 in 2009, but have remained under 20 per year for 11 out of the 14 years sampled. Even with Iran instituting a 15 ng/g maximum allowable aflatoxin regulation in 2003, the number and proportion of consignments rejected for excessively high aflatoxins has remained higher than those from the US. Between 2003 and 2005 inclusive, Iran and the US exported similar amounts of pistachios to the EU; yet the number of rejections over the three-year span for excessively high aflatoxin levels was 477 for Iran vs. 9 for the US. As of 2010, the number of Iranian pistachio consignment rejections remained higher than the US, though Iran exported 35,000 fewer tons than the US to the EU. It appears as though the US crop has remained a more viable option because of lower aflatoxin levels, and the EU has increasingly accepted pistachios from the US.

A 2007 report by the Joint FAO/WHO Expert Committee on Food Additives [12] (JECFA) obtained 1849 pistachio samples from Iran to be tested for aflatoxin. The mean level of aflatoxin detected in these samples was 54 ng/g. As of 2007, aflatoxin regulatory limits for pistachios ranged from 4 ng/g to 35 ng/g around the world. JECFA estimates that the proportion of rejected pistachio consignment samples from Iran would range from 40% in countries with MLs at 20 ng/g, to 60% in countries, like those in the EU, with MLs at 4 ng/g. The high mean level of aflatoxin reported by JECFA was likely caused by several samples containing extremely high levels of aflatoxins.

No reports or publications were found in the publicly available literature that estimated aflatoxin levels in pistachios produced in the United States. However, the EU RASFF database has reported aflatoxin levels in rejected consignments since 2003; from which it is possible to infer relative pistachio quality from exporters to the EU, including the US. The mean level of aflatoxin reported in rejected consignments sent from the US to the EU between 2003 and 2011 was found to be 24 ng/g, whereas the mean rejected level in Iranian pistachios was 63 ng/g. The mean total aflatoxin level of US to EU rejected crops is less than half of the Iranian crops.

**Figure 7:**
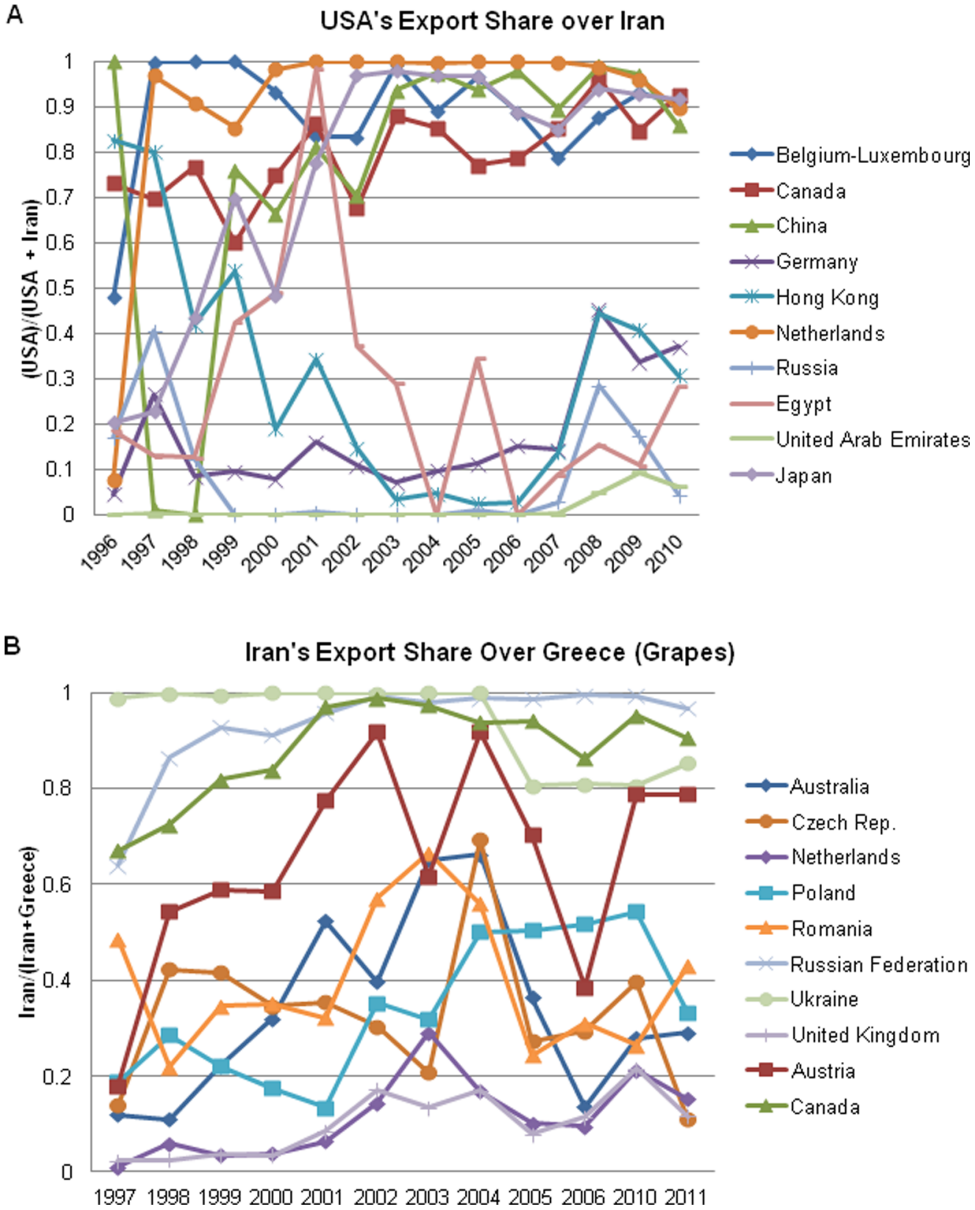
Market segregation and grape control crop analysis. **A)** Market segregation for the top ten global importers of pistachios, as a function of the ratio of total US exports to total exports from both Iran and the US. In the 1990 s until 2003, no distinct market segregation is apparent. In 2004, the US and Iran are exporting pistachios to distinctly different countries. **B)** Lack of market segregation for grape exports from Iran and Greece. Serving as a control crop, ten of the top markets for Iran and Greece were followed from 1997–2011. Unlike the case with pistachios, in which market segregation occurs possibly from aflatoxin regulation, no market segregation is evident in Iranian vs. Greek grape exports.

**Figure 7A** demonstrates the market segregation of global pistachio trade, with nations with stricter aflatoxin standards importing primarily from the US, and nations with more relaxed or nonexistent aflatoxin standards importing primarily from Iran. There appeared to be little or no market segregation in the mid to late 1990s, with most countries importing equally from Iran and the US. Russia and Germany appeared to import the majority of its pistachios from Iran over the past 15 years, whereas Canada and Belgium imported mainly from the US. In 2003, when the EU set its most stringent limit for aflatoxins in pistachios, the market began to segregate with the Netherlands, Belgium, Canada, China, and Japan importing 70% or more of its crop from the US, while Russia, Egypt, UAE, Hong Kong and Germany imported primarily from Iran. When comparing the number of rejections by the EU (Figure 6B), the market appeared to respond accordingly by segregating, with more strict countries importing from the US and more lenient countries importing from Iran. Among these top ten pistachio-importing countries, China is the only country to which the US exports without an aflatoxin regulation. The remaining four countries import primarily or exclusively from the US have aflatoxin regulations at 15 ng/g or stricter. On the other hand, Iran exports to Egypt and the UAE, which have no regulations, to Hong Kong which has a regulation on aflatoxin at 15 ng/g, Russia at 10 ng/g, and Germany at 4 ng/g. **Figure 7B** summarizes grape export data used as a control, to bolster the hypothesis that the pistachio market segregation occurred based upon aflatoxin standards rather than other policy factors, including political ones. Unlike the pistachio data where a split in the markets was apparent around 2003, no obvious segregation of markets between Iran and Greece was seen in the same year. It appeared that only a few countries import only from one country, while most countries imported grapes at varying levels from each country over the 15-year period. Grapes, unlike pistachios, are not subject to aflatoxin regulations.

**Figure 8:**
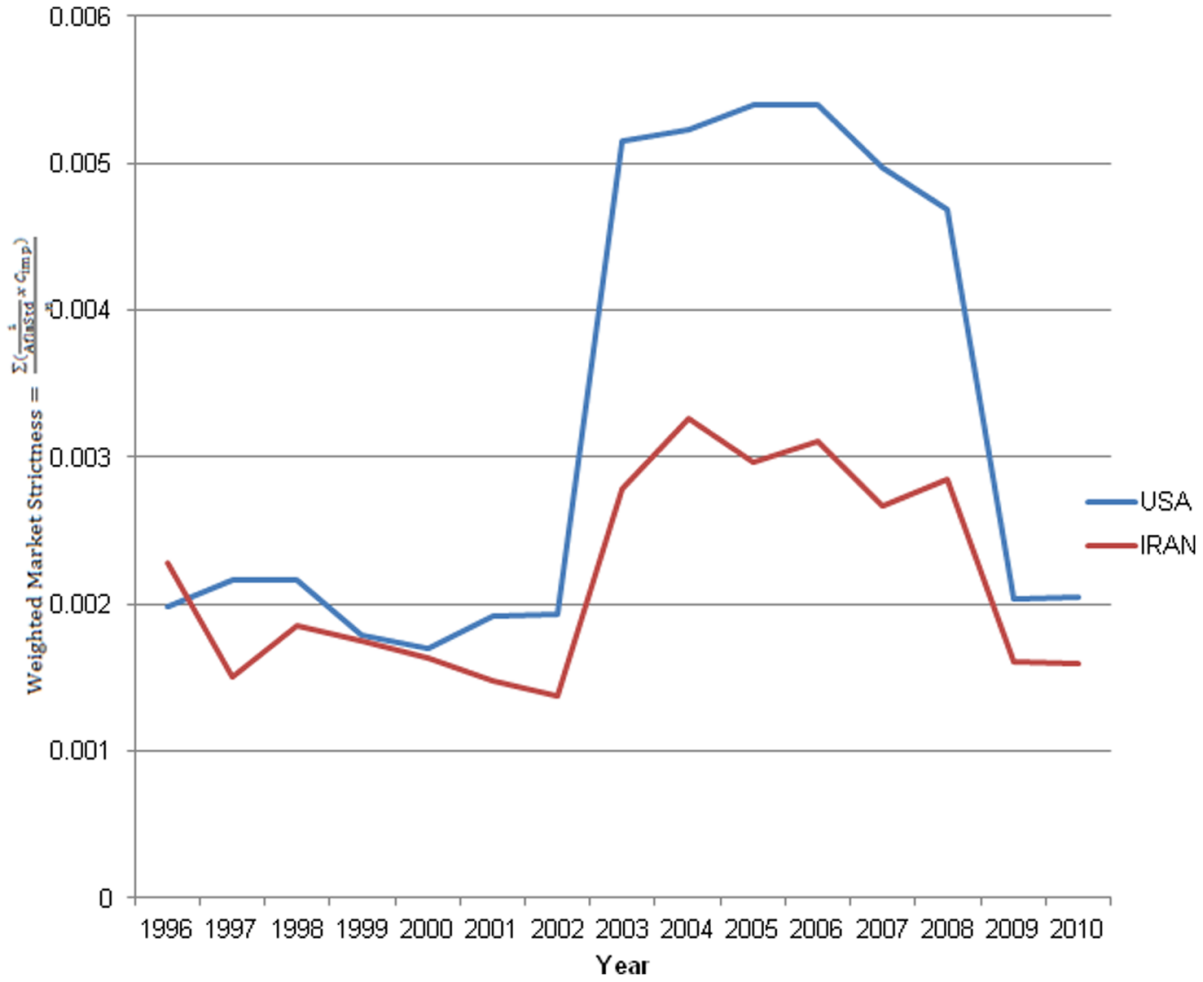
Market segregation as a function of importing nations’ aflatoxin standards over time. The US has exported to countries with stricter aflatoxin standards since 1996; however, until 2003, a distinct difference was not apparent. In 2003, the US was exporting to more countries with stricter aflatoxin standards than Iran. The mean was calculated by summing the inverse of the importing countries aflatoxin standard multiplied by the exports from the US and Iran.

As a final assessment to determine if differences between pistachio-trade patterns of US and Iran exist when the amount of pistachio exports to each country was weighted with the importing nations’ aflatoxin standards (**Figure 8**). There appeared to be no difference in exports until 2003, when the markets began segregating with the US exporting to countries with stricter aflatoxin standards. Iran, continuing to export a larger pistachio crop, was exporting to countries with less strict regulations. After 2009, when the EU relaxed its tree nut standard to 10 ng/g, the market segregation began to diminish, although the US is still the EU’s main pistachio source.

## Discussion

The time-series network modeling analyses conducted in this study suggest that the global pistachio market is segregated based on aflatoxin regulations worldwide, with the top exporters, the United States and Iran, exporting to nations with strict standards and relaxed/non-existent standards, respectively. Iran once dominated the global pistachio market; however, in recent years, the US has increased its pistachio exports substantially. Since 2003, the US has been exporting the majority of it pistachios to countries with stricter aflatoxin standards than Iran. Regardless of the amount produced and viability of the crop, Iran continues to trade with countries with relaxed regulation levels, or no aflatoxin regulations at all.

Since the implementation of the European Union’s RASFF system for tracking consignment rejections due to aflatoxin, rejections by the EU of Iranian pistachios have greatly exceeded those of US pistachios. Between 2003 and 2005, the US and Iran exported similar amounts of pistachios to the EU, but the number of rejected consignments for excessively high aflatoxin in pistachios was substantially higher for Iran than for the US (477 vs. 9). After 2005, Iran pistachio rejections by the EU decreased, but this is likely due to the decrease in Iranian imports and increase of US pistachio imports to the EU.

Clear evidence of market segregation based on aflatoxin regulations started in about 2003. Prior to 2003, the main pistachio-importing countries varied their pistachio imports between the US and Iran. However, as aflatoxin regulations became stricter in certain nations worldwide, the US became the major exporter to countries with strict standards.

Political factors were considered when analyzing results. Since 2006, the United Nations (UN) has imposed multiple sanctions on Iran [22]. In total, six different UN sanctions occurred between 2006 and 2010. Investigating each sanction, no sanctions were placed on food or feed trade between Iran and members of the UN. The sanctions focused on embargoes on arms and assets, which would likely have little or no impact to the global trade of pistachios. Indeed, the grape market (of which Iran is a key exporter) shows no evidence of segregation based on different nations. It is used as a control in this study to demonstrate the potential role of the aflatoxin regulations in contributing to the market segregation seen in global pistachio trade.

Due to segregation in the global market, not only for pistachios but also for maize and other aflatoxin-contaminated commodities [9], many economic and health issues may arise [20]. First, strict aflatoxin standards mean that less developed nations will export their best crops to avoid economic losses, but in turn be subject so consuming the highest contaminated crops themselves. Second, due to varying aflatoxin regulations in each country, even the best crops may be rejected resulting in large economic losses. Third, even if a rejected consignment can be returned to the country attempting to export, the cost of demurrage fees is substantial, and vulnerable populations may be exposed to higher levels of aflatoxin, resulting in adverse health effects. In many cases, it is low-income importing nations that have more relaxed or non-existent aflatoxin regulations, predisposing populations who are already at risk of various health effects from inadequate diets to higher levels of risk.

In summary, social network models show that pistachio trade patterns have changed globally over the past 15 years, with aflatoxin regulations likely playing a key role in the changing patterns of trade. Iran once dominated the global market individually, but now must compete with the US to be the world’s top exporter of the crop. Aflatoxin regulations play a part in organizing the global trade of the crop, with the US exporting to countries with stricter aflatoxin standards. Whether it is to protect human health or reduce economic losses, countries are increasingly importing pistachios from the US, especially those countries with strict maximum levels.
